# Cysticercosis and neurocysticercosis in Eastern Zambia: A cross-sectional, community-based study

**DOI:** 10.1016/j.ijregi.2026.101018

**Published:** 2026-09-21

**Authors:** Gideon Zulu, Kabemba Evans Mwape, Tamara Margarete Welte, Dominik Stelzle, Martin Simuunza, Philipp Prucker, Alex Hachangu, Wilbroad Mutale, Maxwell Masuku, Charlotte Ruether, Chummy Sikalizyo Sikasunge, Isaac Khozozo Phiri, Andrea Sylvia Winkler

**Affiliations:** 1 Ministry of Health, Government of the Republic of Zambia, Lusaka, Zambia; 2 Department of Clinical Studies, School of Veterinary Medicine, University of Zambia, Lusaka, Zambia; 3 Department of Neurology, TUM University Hospital and Center for Global Health, School of Medicine and Health, Technical University of Munich (TUM), Munich, Germany; 4 Epilepsy Center, Department of Neurology, University Hospital Erlangen, Erlangen, Germany; 5 Department of Disease Control, School of Veterinary Medicine, University of Zambia, Lusaka, Zambia; 6 Department of Diagnostic and Interventional Radiology, Klinikum rechts der Isar, School of Medicine and Health, TUM University Hospital, Technical University of Munich, Munich, Germany; 7 Department of Public Health, School of Medicine, University of Zambia, Lusaka, Zambia; 8 Department of Radiology, Faculty of Medicine, Technical University of Munich, Munich, Germany; 9 Department of Para-clinical Studies, School of Veterinary Medicine, University of Zambia, Lusaka, Zambia; 10 Department of Community Medicine and Global Health, Institute of Health and Society, Faculty of Medicine, University of Oslo, Oslo, Norway; 11 Department of Global Health and Social Medicine, Harvard Medical School, Boston, MA, USA

**Keywords:** *Taenia solium*, Cysticercosis, Neurocysticercosis, Epilepsy, Zambia

## Abstract

**Objective:**

This study aimed to estimate the seroprevalence of cysticercosis and the prevalence of neurocysticercosis (NCC) among cysticercosis-positive participants, and to describe their clinical and radiological manifestations in rural Zambia.

**Methods:**

A cross-sectional study was conducted between June 2019 and December 2022 in the Eastern Province of Zambia. The serum antigen enzyme-linked immunosorbent assay (Ag-ELISA) and Cysticercosis immunoglobulin G (IgG) Western blot were used to detect cysticercosis. All cysticercosis (CC)-positive participants received further clinical work-up and cerebral computed tomography (CT) examination.

**Results:**

Out of 2876 serum samples examined, 70 (2.4%) [95% confidence interval (CI) = 1.9-3.1%] tested positive only on Ag-ELISA, 149 (5.2%) [95% CI = 4.4-6.1%] tested positive only on the Western blot and 35 (1.2%) [95% CI = 0.9-1.7%] were positive on both the antigen and antibody tests, yielding a total of 254 (8.8%) [95% CI = 7.8-9.9%] positive on at least one serological test. More males than females were CC-positive (10.6% versus 7.6%, *P* = 0.005); seropositivity increased with age (*P* = 0.046) and differed between villages (*P* = 0.001). Among the CC-positive participants, 236/254 (93%) had a clinical work-up and cerebral CT examination, of whom 80/236 (34%) [95% CI = 28-40%] had NCC. Overall, neurological deficits were rare, the most common being cranial nerve abnormalities (23/236, 10%). However, headache and epileptic seizures were more frequently reported among participants with NCC than among those without NCC [37/80 (46%) versus 52/156 (33%) for headache (*P* = 0.05) and 13/80 (16%) versus 12/156 (8%) for epileptic seizures (*P* = 0.04)]. Regarding NCC lesions in people with epileptic seizures, 53% (52/99) and 96% (95/99) of all lesions were in the brain parenchyma and inactive (calcified), respectively. For people with headache, 68% (139/205) and 83% (170/205) of all lesions were in the parenchyma and inactive, respectively.

**Conclusion:**

Our findings demonstrate that cysticercosis and NCC are prevalent in this rural community in the Eastern Province of Zambia. Most of the patients with NCC had calcified lesions, and people with NCC more often had epileptic seizures and headache than those without NCC; only a few people had neurological deficits.

## Highlights

• This study highlighted the clinical and radiological features of cysticercosis-positive participants in the Eastern Province of Zambia.
• The common manifestations of neurocysticercosis in community settings are epileptic seizures and headaches.
• Epileptic seizures and headache are commonly found in patients with neurocysticercosis and calcified lesions.
• There is a need for context-specific neurocysticercosis management guidelines focusing on resource-limited settings.

## Background

Cysticercosis (CC) is an infection with the larval form (cysticerci) of the pork tapeworm, *Taenia solium*. Neurocysticercosis (NCC) occurs when the cysticerci of *T. solium* lodge in the central nervous system. NCC has significant clinical and public health implications because humans may develop multiple neurological manifestations including epilepsy, severe chronic headaches and focal neurologic deficits [[1], [2], [3]]. The neurological signs and symptoms that are associated with NCC are diverse and depend on the number, size, location, and stage of NCC lesions as well as the presence of inflammation in addition to host factors [4]. Depending on the location of cysticerci, two forms of NCC are recognized: parenchymal (within the brain tissue) and extraparenchymal (commonly in the subarachnoid spaces at the base of the skull or around the convexities and the ventricles) [5]. Parenchymal NCC, if symptomatic, commonly presents with epileptic seizures, is relatively easy to treat, and has a relatively good prognosis if the number of lesions is not too large [5,6]. Extraparenchymal NCC, on the other hand, is more difficult to treat, especially if the lesions are either in the ventricles or in the basal subarachnoid cisterns [7]. Patients with ventricular cysts commonly present with signs and symptoms of increased intracranial pressure such as severe progressive headache and, in general, have a poor prognosis [8].

Within Africa, it is only recently that some light has been shed on the prevalence, as well as clinical and neuroradiological characteristics of people with NCC [1,[9], [10], [11]]. However, most of these studies were hospital-based or were conducted in neurologically symptomatic individuals, mainly in people with epilepsy. In Zambia, the prevalence of NCC among people with epilepsy was found to be more than 50% in a highly endemic area for *T. solium* [12], although this was a rather small study based on 56 people with epilepsy who were selected from *T. solium*-endemic communities. However, it is well known that most people with NCC are clinically asymptomatic [2]; hence, individuals need to be recruited independently of the presence of neurological signs and symptoms, best within large community-based studies. These data are essential to accurately estimate the burden of NCC, including its clinical and neuroradiological presentation, and to tailor clinical management strategies as well as public health interventions [3]. This study therefore aimed to estimate the community-based seroprevalence of CC and the prevalence of NCC irrespective of the presence or absence of neurological signs and symptoms in a population of almost 3000 people of the Eastern Province of Zambia, as well as to understand their clinical and radiological manifestations of NCC.

## Methods

### Study site

The study was conducted between June 2019 and December 2022 in Chiparamba, an area located at 13.6375° S, 32.3108° E and about 50 km from the central business district of Chipata in the Eastern Province of Zambia. Several studies on *T. solium* had been conducted in other regions of the Eastern Province [[11], [12], [13], [14], [15]] but not in Chipata District. The Chiparamba area was selected based on reports from the Chipata District Health Office of high numbers of people with epileptic seizures (PWEs) presenting to Chiparamba Rural Health Centre (RHC), the high numbers of freely roaming pigs, and reports of cysts observed in pigs slaughtered for sale.

### Study design and participant selection

A community-based cross-sectional study was conducted. This study was part of the *Taenia solium* Experimental Pig Infection Model (TEPIM) project “Establishment and application of a *T. solium* experimental pig infection model and investigation of environmental factors associated with transmission of *T. solium* in endemic villages of the Eastern and Southern provinces of Zambia.” The human part of the study involved the collection of tapeworm proglottids for the experimental pig infection model. Sample size estimation was thus based on the number of *T. solium* proglottids required for the experimental pig infection study. A total of 40 proglottids were required. At a reported taeniasis prevalence of 0.3% by microscopic examination [14] and assuming that two gravid proglottids were to be obtained from each tapeworm collected, a total of 6668 people were to be tested for taeniasis from two districts, Chipata and Gwembe. Because the experimental infection study was discontinued due to logistical reasons and the Gwembe District site was not sampled, a sample size of 3334 participants (i.e., half of the total sample) was targeted to estimate the prevalence of taeniasis and CC from Chipata District. To reach this number, a list of all the villages with their populations under the Chiparamba RHC catchment was obtained, and 25 villages were randomly selected for the study.

The process involved conducting a sensitization meeting with all villagers during which *T. solium* infections were discussed using the *T. solium* life cycle. Prevention measures for *T. solium* infections were also discussed during these sensitization meetings. After the objectives of the study had been explained, all participants from that village were invited to freely participate in providing stool and blood samples for analysis. All participants living in the study area, willing and able to provide informed consent, and aged 10 years or older, were eligible to participate in the study and were invited to a central place within each village for screening. Participants were excluded if the listed inclusion criteria were not met or if they were seriously ill (unable to engage in the normal activities of daily living without assistance because of their illnesses) at the time of the study.

### Laboratory analysis of blood samples

The serum antigen enzyme-linked immunosorbent assay (Ag-ELISA) (apDia Belgium) commercial kit was used to detect cysticercal antigens, and the CC immunoglobulin G (IgG) Western blot (LDBIO Diagnostics; hereafter referred to as Western blot) was used to detect cysticercal antibodies from collected blood samples [16,17]. An individual was classified as positive for CC if they tested positive on either the serum Ag-ELISA or the Western blot.

### Clinical assessment

All participants testing positive for CC antigens and/or antibodies received a clinical work-up, which involved performing a general medical and neurological examination as well as cerebral computed tomography (CT) with and without contrast (Figure 1). Demographic and clinical data including information on seizure history and antiseizure medication were also collected. Information on the nature and frequency of headache was also collected. The questions on headache referred to headache that was severe enough (severe headache) to interfere with normal daily activity. The recall period for headache symptoms referred to headache within the last 1 year. A neurological examination was performed by a medical doctor.

**Figure 1:**
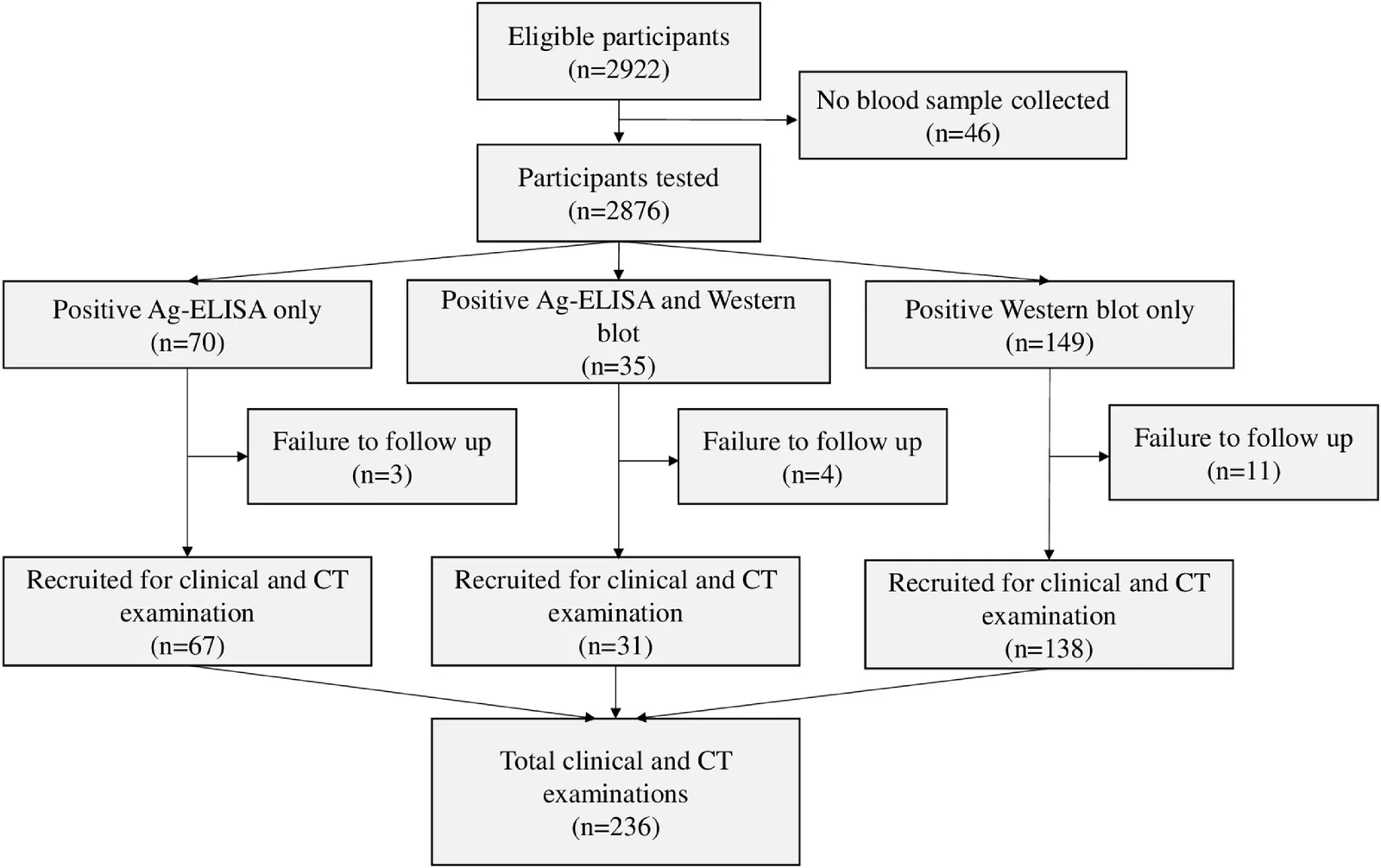
Participant selection for clinical and computed tomography (CT) examination.

### Radiological assessment and neurocysticercosis diagnosis

Brain imaging with and without contrast was performed at Chipata Central Hospital using a Neusoft CT scanner with 16 slices per revolution and a slice thickness of 1.5 mm. The CT scans were evaluated by neuroradiologists (Charlotte Ruether and Philipp Prucker). For every CT examination, the number, location, and stage of the NCC lesions were recorded. The stage of NCC lesions was categorized as active (vesicular, colloidal vesicular, granular nodular) or inactive (calcified) [18,19]. The location of the lesions was further classified as parenchymal (frontal lobe, temporal lobe, parietal lobe, occipital lobe, cerebellum, brainstem) or extraparenchymal (intraventricular or subarachnoid). The final diagnosis was arrived at and classified as definite or probable NCC according to the neuroimaging and clinical/exposure criteria as suggested by the 2017 revised Del Brutto et al. criteria [20].

### Data analysis

Statistical analyses were performed using IBM SPSS Statistics (version 23). Numbers and proportions were presented for binary variables, whereas continuous data were presented as the median and interquartile range (IQR). Associations between categorical variables were determined using either the chi-square test or Fisher’s exact test. Binary logistic regression was used to determine the predictors for seropositivity to CC. Differences in clinical characteristics between participants with NCC and those without NCC were also analyzed using the chi-square test or Fisher’s exact test for categorical variables, and Mann-Whitney *U* tests for continuous data. Comparisons by stage of NCC (active stage versus inactive stage) and serological test positivity as well as by the presence of epileptic seizures (PWEs and people without epileptic seizures [PWnE]) and radiological characteristics were made using the same tests. All statistics were considered significant at *P* < 0.05.

## Results

Serum samples were collected from 2876 participants recruited in the study. The majority were female (1667, 58.0%). The largest age group (1233, 42.9%) comprised participants aged 10-20 years. The median age was 24 years (IQR = 14-39) (Table 1).

**Table 1:** Demographic characteristics of participants (*n* = 2876).

Description	Variable	Number (%)
Sex	Male	1209 (42.0)
	Female	1667 (58.0)
Age group (years)	10-20	1233 (42.9)
	21-40	931 (32.4)
	41-60	527 (18.3)
	61-80	162 (5.6)
	81-100	23 (0.8)
Age	Median in years (IQR)	24 (14-39)

Abbreviation: IQR, interquartile range.

### CC

Of the 2876 samples tested, 70 (2.4%) [95% confidence interval (CI) = 1.9-3.1%] tested positive only on Ag-ELISA, 149 (5.2%) [95% CI = 4.4-6.1%] tested positive only on the Western blot and 35 (1.2%) [95% CI = 0.9-1.7%] were positive on both the antigen and antibody tests, giving a total of 254 (8.8%) [95% CI = 7.8-9.9%] positive on at least one serological test (Table 2). Sex, age, and village were significantly associated with CC seropositivity in at least one serological test (Table 2, Additional file Table S1). Males more often had a positive serologic test (*P* = 0.005) than females, and seropositivity increased with age group (*P* = 0.046). Seropositivity differed across the 25 included villages (*P* = 0.001), and only one village (Chimutangati) had nobody (0/31) testing serologically positive for CC. The results were similar in the regression model, with sex and age being significant predictors of at least one positive serologic test result (Additional file Table S2).

**Table 2:** Cysticercosis seroprevalence by age and sex.

Description		Total	At least one serological test positive *n* (%)	Ag-ELISA positive only *n* (%)	Western blot positive only *n* (%)	Ag-ELISA and Western blot positive *n* (%)	*P*-value^a^
		2876	254	70	149	35	
Sex	Male	1209	128 (10.6)	35 (2.9)	73 (6.0)	20 (1.7)	0.005
	Female	1667	126 (7.6)	35 (2.1)	76 (4.6)	15 (0.9)	
Age	10-20	1233	98 (7.9)	26 (2.1)	62 (5.0)	10 (0.8)	0.046
	21-40	931	81 (8.7)	23 (2.5)	45 (4.8)	13 (1.4)	
	41-60	527	54 (10.2)	16 (3.0)	29 (5.5)	9 (1.7)	
	61-80	162	15 (9.3)	4 (2.5)	8 (4.9)	3 (1.9)	
	81-100	23	6 (26.1)	1 (4.3)	5 (21.7)	0 (0.0)	

a *P*-value for the comparison of sex/age with the variable “at least one serological test positive.” Abbreviations: Ag, antigen; ELISA, enzyme-linked immunosorbent assay.

### Neurocysticercosis

From the 254 CC seropositive participants, 18 (7%) were lost to follow-up, while 236 (93%) had a further clinical work-up and cerebral CT examination. Eighty (34%) [95% CI = 28-40%] of these had NCC, 48/138 (35%) [95% CI = 28-43%] among those positive on only the Western blot, 12/67 (18%) [95% CI = 11-29%] among those positive on only Ag-ELISA, and 20/31 (65%) [95% CI = 47-79%] among those positive on both the Western blot and Ag-ELISA (Table 3). All participants with NCC had one major neuroimaging criterion (presence of NCC-suggestive lesions), one major clinical/exposure criterion (positive CC serology), and one minor clinical/exposure criterion (coming from a CC-endemic area). Thus, all participants with NCC were diagnosed to have definite NCC according to the revised Del Brutto et al. criteria [20].

**Table 3:** Prevalence of neurocysticercosis in the Chiparamba area of Chipata District.

Description	Total	Western blot only	Ag-ELISA only	Both Western blot and Ag-ELISA
Serology positive results and CT examination	236	138	67	31
Participants with NCC	80	48	12	20
Certainty of diagnosis (definite versus probable)	Definite	48	12	20
	Probable	0	0	0
NCC prevalence among CC-positive participants (95% CI)	34% (28-40)	35% (28-43)	18% (11-29)	65% (47-79)

Abbreviations: Ag, antigen; CC, cysticercosis; CI, confidence interval; CT, computed tomography; ELISA, enzyme-linked immunosorbent assay; NCC, neurocysticercosis.

Participants with NCC did not differ significantly from those without NCC with respect to sex, age, religion, level of education, or occupation (Table 4).

**Table 4:** Demographic and clinical characteristics among cysticercosis-positive participants with and without neurocysticercosis in the Chiparamba area of Chipata District.

Description	Variable	Overall *n* (%)	NCC *n* (%)	No NCC *n* (%)	*P*-value
N		236	80	156	
Participant sex	Male	122 (51.7)	38 (47.5)	84 (53.8)	0.36
	Female	114 (48.3)	42 (52.5)	72 (46.2)	
Age	Median in years (IQR)	26.5 (14-43)	26.5 (13-44)	25.5 (14-42)	0.76
Age range (years)	10-20	85 (36.0)	26 (32.5)	59 (37.8)	0.52
	21-40	83 (35.2)	27 (33.8)	56 (35.9)	
	41-60	48 (20.3)	17 (21.2)	31 (19.9)	
	61-80	15 (6.4)	8 (10.0)	7 (4.5)	
	81-100	5 (2.1)	2 (2.5)	3 (1.9)	
Religion	Christian	217 (91.9)	71 (88.8)	146 (93.6)	0.39
	Muslim	6 (2.5)	4 (5.0)	2 (1.3)	
	None	13 (5.5)	5 (6.2)	8 (5.1)	
Highest level of education	None	37 (15.7)	14 (17.5)	23 (14.7)	0.84
	Primary school	161 (68.2)	54 (67.5)	107 (68.6)	
	Secondary school	38 (16.1)	12 (15.0)	26 (16.7)	
Main occupation	None	11 (4.7)	4 (5.0)	7 (4.5)	0.87
	Farmer	163 (69.1)	55 (68.8)	108 (69.2)	
	Student	57 (24.2)	19 (23.8)	38 (24.4)	
	Formal employment	1 (0.4)	0 (0.0)	1 (0.6)	
	Trader	3 (1.3)	1 (1.2)	2 (1.3)	
	Bicycle taxi	1 (0.4)	1 (1.2)	0 (0.0)	
**Clinical characteristics**					
***Severe headache***	Yes	89 (37.7)	37 (46.2)	52 (33.3)	0.05
Frequency of headaches	More than once a month	38 (42.7)	15 (40.5)	23 (44.2)	0.93
	Less than once a month	45 (50.6)	20 (54.1)	25 (48.1)	
	Cannot remember	5 (5.6)	2 (5.4)	3 (5.8)	
	Every day	1 (1.1)	0 (0.0)	1 (1.9)	
Last time had a headache	More than a month ago	55 (61.8)	20 (54.1)	35 (67.3)	0.42
	Cannot remember	19 (21.3)	10 (27.0)	9 (17.3)	
	Currently have	15 (16.9)	7 (18.9)	8 (15.4)	
***Epileptic seizures***	Yes	25 (10.6)	13 (16.2)	12 (7.7)	0.04
Age of all participants with seizures in years	Median (IQR)	30 (20-52)	39 (24-62)	14 (10-19)	0.04
Age of seizure onset in years	Median (IQR)	15 (5-22)	15 (12-24)	14 (10-18)	0.89
Frequency of seizures	More than once a month	12 (48.0)	5 (38.5)	7 (58.3)	0.76
	Less than once a month	10 (40.0)	6 (46.1)	4 (33.3)	
	Cannot remember	3 (12.0)	2 (15.4)	1 (8.3)	
Last time had a seizure	More than a month ago	16 (64.0)	8 (61.5)	8 (66.7)	0.58
	Cannot remember	2 (8.0)	2 (15.4)	0 (0.0)	
	Few days to weeks ago	7 (28.0)	3 (23.1)	4 (33.3)	
Risk factors	Family member with epilepsy	4 (16.0)	2 (15.4)	2 (16.7)	1.00
	History of head injury with loss of consciousness	1 (4.0)	0 (0.0)	1 (8.3)	0.73
Impact of seizure	Injured because of seizure	12 (48.0)	8 (61.5)	4 (33.3)	0.16
	*Fell to the ground*	9 (75.0)	6 (75.0)	3 (75.0)	>0.99
	*Got burned by fire*	3 (25.0)	2 (25.0)	1 (25.0)	
Seizure treatment	Yes	22 (88.0)	11 (84.6)	11 (91.7)	>0.99
Antiseizure medication	*Carbamazepine*	17 (77.3)	8 (72.7)	9 (81.8)	>0.99
	*Traditional medicine*	3 (13.6)	2 (18.2)	1 (9.1)	
	*Phenobarbital*	1 (4.5)	0 (0.0)	1 (9.1)	
	*Cannot remember*	1 (4.5)	1 (9.1)	0 (0.0)	
***Ever had skin nodules***	Yes	5 (2.1)	4 (5.0)	1 (0.6)	0.08
**Neurological examination**					
Cranial nerve abnormalities		23 (9.7)	9 (11.2)	14 (9.0)	0.58
Muscle strength deficits		3 (1.3)	1 (1.2)	2 (1.3)	>0.99
Muscle tone abnormalities (hypertonia)		2 (0.8)	1 (1.2)	1 (0.6)	>0.99
Deep tendon reflex abnormalities		3 (1.3)	2 (2.5)	1 (0.6)	0.27
Extrapyramidal abnormalities		2 (0.8)	1 (1.2)	1 (0.6)	>0.99
Gait abnormalities		4 (1.7)	2 (2.5)	2 (1.3)	0.61
Coordination abnormalities		2 (0.8)	1 (1.2)	1 (0.6)	>0.99
Sensation abnormalities		0 (0.0)	0 (0.0)	0 (0.0)	NA
Mental state abnormalities		2 (0.8)	1 (1.2)	1 (0.6)	>0.99
**Serological test positivity**					
*Western blot only*		138 (58.5)	48 (60)	90 (57.7)	0.781
*Ag-ELISA only*		67 (28.4)	12 (15)	55 (35.3)	0.001
*Both Western blot and Ag-ELISA*		31 (13.1)	20 (25)	11 (7.0)	0.000

Abbreviations: Ag, antigen; ELISA, enzyme-linked immunosorbent assay; IQR, interquartile range; NA, Not applicable; NCC, neurocysticercosis.

### Neurological signs and symptoms

Severe headache was more frequently reported among participants with NCC than among those without NCC [37/80 (46%) versus 52/156 (33%), *P* = 0.05], but there was no significant difference in terms of frequency of the headache episodes between the two groups (*P* = 0.93). Epileptic seizures were more common among participants with NCC [13/80 (16%) versus 12/156 (8%), *P* = 0.04]. The median age of participants with seizures was much higher among those with NCC [39 years (24-62) versus 14 years (4-19), *P* = 0.04], but there was neither a statistically significant difference in the median age of seizure onset (*P* = 0.89) nor in seizure frequency (*P* = 0.76) between those with NCC and those without NCC. Though neurological deficits were rare among all participants examined, the most common were cranial nerve abnormalities (23/236, 10%). Regarding mental state examination, only two participants displayed behavioral and cognitive impairment, one with NCC and the other without NCC. Overall, there were no differences between participants with NCC and those without NCC in terms of neurological examination findings (Table 4). A summary of the abnormal neurological examination findings is shown in Additional file Table S3.

### Neurocysticercosis lesions on neuroimaging

Among the 80 participants with NCC, 10 (13%) had active-stage lesions only, 56 (70%) had inactive (calcified) lesions only, and 14 (18%) had both active and inactive lesions (Table 5). Of the total number of lesions, only 22% (78/355) were lesions in the active stage, with each participant having a median of 4 (1-8) active lesions, the majority (53/78, 68%) of which were located in the parenchyma. Overall, 69% (190/277) of all inactive lesions were located in the parenchyma with a median number of 1 (1-3) lesions per participant.

**Table 5:** Prevalence and radiological characteristics of neurocysticercosis among cysticercosis-positive participants (*n* = 80).

Description	Total	Western blot only	Ag-ELISA only	Both Western blot and Ag-ELISA
**Participants with NCC**	80	48	12	20
Participants with active lesions only (%)	10 (12.5)	7 (14.6)	1 (8.3)	2 (10.0)
Participants with calcified lesions only (%)	56 (70.0)	37 (77.1)	10 (83.3)	9 (45.0)
Participants with active and calcified lesions (%)	14 (17.5)	4 (8.3)	1 (8.3)	9 (45.0)
**NCC-typical lesions**				
Number	355	228	22	105
Mean per patient	4.4	4.8	1.8	5.2
Median per patient [IQR]	2 (1-4)	1 (1-4)	1 (1-2)	2 (1-4)
**Active NCC-typical lesions (vesicular, colloidal, granular)**				
Number of active lesions	78	31	3	44
Mean per patient	3.2	2.8	1.5	4.0
Median per patient	4 (1-8)	2 (0.5-6.5)	0.5 (0-1.2)	2 (0-9)
Active lesions among all lesions (%)	22.0	13.6	13.6	41.9
Median proportion of active lesions per patient (%) [IQR]	5.1 (1.3-10%)	6.5 (1.6-21%)	16. (0-42%)	4.5 (0-20%)
**Location of active lesions (parenchymal/extraparenchymal)**				
Parenchymal (N)	53	21	2	30
Extraparenchymal (N)	25	10	1	14
Extraparenchymal lesions (%)	32.1	32.3	33.3	31.8
**Inactive NCC-typical lesions (calcifications)**				
Number of calcified lesions	277	197	19	61
Mean per patient	4.0	4.8	1.7	3.4
Median per patient	1 (1-3)	1 (1-3)	1 (1-3)	1 (1-3)
Inactive lesions among all lesions (%)	78.0	86.4	86.4	58.1
Median proportion of inactive lesions per patient (%) [IQR]	0.36 (0.36-1.08)	0.51 (0.51-1.52)	5.26 (5.26-15.79)	1.64 (1.64-4.92)
**Location of inactive lesions (parenchymal/extraparenchymal)**				
Parenchymal (N)	190	127	14	49
Extraparenchymal (N)	87	70	5	12
Extraparenchymal lesions (%)	31.4	35.5	26.3	19.7

Abbreviations: Ag, antigen; ELISA, enzyme-linked immunosorbent assay; IQR, interquartile range; NCC, neurocysticercosis.

### Neurocysticercosis lesions and their association with serological results

The highest number of lesions (228/355, 64%) was recorded among participants positive on the Western blot only, followed by participants positive on both the Western blot and Ag-ELISA (105/355, 30%) and the lowest number (22/355, 6%) was recorded among participants positive on Ag-ELISA only. Overall, participants had a median of 2 (1-4) lesions. The highest number of active-stage lesions was found among participants positive on both the Western blot and Ag-ELISA (44/78, 56%), but the median proportion of active-stage lesions per person was highest [17% (0-42)] among those positive on Ag-ELISA only. The proportion of extraparenchymal lesions among all active-stage lesions was similar (∼32%) among all participants regardless of the test result. The highest number of inactive-stage lesions was found among participants positive on the Western blot only (197/277, 71%). The median number of inactive lesions [1 (1-3)] was similar in all participants regardless of the test result. Overall, 31% of all inactive-stage lesions were located in the extraparenchymal space. Among the 156 participants without NCC, 12 (8%) had abnormal radiological findings on cerebral CT examination (Additional file Table S4).

### Neurocysticercosis lesions and their association with severe headache and epileptic seizures

Of all participants with NCC, 37 (46%) reported experiencing severe headache (Table 6). These had a total of 205 lesions, most (170/205, 83%) of which were inactive. Those with NCC but no headache (43/80, 54%) had overall 150 lesions of which 107 (71%) were inactive. Overall, the median number of lesions did not differ significantly between those with NCC and headache and those with NCC without headache [2 (1-3) and 3 (1-5), *P* = 0.44]. There was no significant difference either in the proportion of participants with active-stage lesions or in the median number of active lesions or calcifications per patient between people with NCC and headache and those without headache. Among patients with NCC and headache, the majority (21/37, 57%) had lesions in the brain parenchyma, whereas among those without headache, the largest group (18/43, 42%) had lesions in both the parenchyma and extraparenchyma. However, the proportion of lesions in the parenchyma among those with NCC and headache (68%) and those without headache (69%) was similar.

**Table 6:** Neurocysticercosis lesions among people with headache (*n* = 37) and people without headache (*n* = 43).

Description	Variable	Headache with NCC	No headache with NCC
		***n* (%)**	***n* (%)**
All participants	Number	37 (46.3)	43 (53.8)
	Number of lesions	205	150
	Mean per participant	5.5	3.5
	Median per participant [IQR]	2 (1-3)	3 (1-5)
Participants with active lesions	Number of participants	11/37 (29.7)	13/43 (30.2)
	Number of lesions	35	43
	Mean per participant	3.2	3.3
	Median per participant [IQR]	1 (1-4)	2 (1-5)
Participants with calcifications	Number of participants	33/37 (89.2)	37/43 (86.0)
	Number of lesions	170	107
	Mean per participant	5.2	2.9
	Median per participant [IQR]	1 (1-3)	3 (1-4)
Participants and location of lesions	Parenchymal only	21/37 (56.8)	17/43 (39.5)
	Extraparenchymal	7/37 (18.9)	8/43 (18.6)
	Both parenchymal/extraparenchymal	9/37 (24.3)	18/43 (41.9)
Location and number of lesions	Parenchymal	139/205 (67.8)	104/150 (69.3)
	Extraparenchymal	66/205 (32.2)	46/150 (30.7)
Parenchymal	Frontal	87 (42.4)	59 (39.3)
	Temporal	22 (10.7)	10 (6.6)
	Parietal	20 (9.8)	19 (12.7)
	Occipital	10 (4.9)	14 (9.3)
	Cerebellum	0 (0.0)	2 (1.3)
	Brainstem	0 (0.0)	0 (0.0)
Extraparenchymal	Subarachnoid	64 (31.2)	42 (28.0)
	Intraventricular	2 (1.0)	4 (2.7)

Abbreviations: IQR, interquartile range; NCC, neurocysticercosis.

Thirteen (16%) of all participants with NCC were PWEs (Table 7). These had a total of 99 lesions, most of which were inactive (95/99, 96%). PWnE with NCC (67/80, 84%) had overall 256 lesions, of which 182 (71%) were inactive. Overall, the median number of lesions did not differ significantly between PWEs and PWnE [3 (1-10) and 1 (1-4), *P* = 0.36]. The proportion of participants with active-stage lesions was similar between PWEs (4/13, 31%) and PWnE (20/67, 30%). However, PWEs had a median of 1 (0) active lesion, while PWnE had a median of 2 (1-5) active lesions, *P* = 0.046. The median number of calcifications per participant was higher among PWEs, 3 (1-11) versus 2 (1-3) among PWnE, though this difference was not statistically significant (*P* = 0.10). Among the PWEs, the largest group (6/13, 46%) had lesions in both the brain parenchyma and the extraparenchymal space, whereas among the PWnE, the largest group (33/67, 49%) had lesions in the parenchyma only. Overall, 53% (52/99) of lesions in PWEs were in the brain parenchyma.

**Table 7:** Neurocysticercosis lesions among people with epileptic seizures (PWEs; *n* = 13) and people without epileptic seizures (PWnE; *n* = 67).

Description	Variable	PWEs with NCC	PWnE with NCC
		***n* (%)**	***n* (%)**
All participants	Number	13 (16.2)	67 (83.8)
	Number of lesions	99	256
	Mean per participant	7.6	3.8
	Median per participant [IQR]	3 (1-10)	1 (1-4)
Participants with active lesions	Number of participants	4/13 (30.8)	20/67 (29.9)
	Number of lesions	4	74
	Mean per participant	1	3.7
	Median per participant [IQR]	1 (0)	2 (1-5)
Participants with calcifications	Number of participants	11/13 (84.6)	59/67 (88.1)
	Number of lesions	95	182
	Mean per participant	8.6	3.1
	Median per participant [IQR]	3 (1-11)	2 (1-3)
Participants and location of lesions	Parenchymal only	5/13 (38.5)	33/67 (49.3)
	Extraparenchymal	2/13 (15.4)	13/67 (19.4)
	Both parenchymal/extraparenchymal	6/13 (46.2)	21/67 (31.3)
Location and number of lesions	Parenchymal	52/99 (52.5)	191/256 (74.6)
	Extraparenchymal	47/99 (47.5)	65/256 (25.4)
Parenchymal	Frontal	32 (61.5)	114 (59.7)
	Temporal	8 (15.4)	24 (12.6)
	Parietal	9 (17.3)	30 (15.7)
	Occipital	2 (3.8)	22 (11.5)
	Cerebellum	1 (1.9)	1 (0.5)
	Brainstem	0 (0.0)	0 (0.0)
Extraparenchymal	Subarachnoid	45 (95.7)	61 (93.8)
	Intraventricular	2 (4.3)	4 (6.2)

Abbreviations: IQR, interquartile range; NCC, neurocysticercosis; PWEs, people with epileptic seizures; PWnE, people without epileptic seizures.

## Discussion

In this study, we assessed the prevalence of CC and the prevalence of NCC among CC-positive people in the Chiparamba area of Chipata District in the Eastern Province of Zambia. We also assessed the clinical characteristics among CC-positive people with and without NCC. Lastly, we analyzed the radiological characteristics of people with NCC.

Our study showed a CC seroprevalence of 3.7% and 6.4% for cysticercal antigens and antibodies, respectively, with great variation between villages. This variation could be attributed to differences in the level of contamination with *T. solium* eggs as well as differences in knowledge, attitudes, and practices relating to *T. solium* infections within the Chiparamba community [21]. More people tested positive for cysticercal antibodies than for cysticercal antigens. This is expected because people who may not have an active infection but have been exposed to the parasite retain the antibodies [22]. Within Zambia and also within the Eastern and Southern African region, higher (5.8-40.8%) CC seroprevalences based on Ag-ELISA have been reported [9]. Higher CC antibody seroprevalences have also been reported in studies conducted in Madagascar (13-14%) [23,24], Tanzania (16.3-45.3%) [25,26], and Rwanda (13.3%) [27]. We cannot explain exactly the reasons for the lower antigen and antibody prevalence in our study, but this could be due to changes in environmental contamination with tapeworm eggs over time, as the taeniasis prevalence on microscopy was also found to be lower (0.4%) in the study area [28]. The CC seroprevalence in people with at least one positive serological test was higher among males than among females. This could be due to pork handling practices among males as they mostly slaughter pigs and handle pork including that infected with cysticerci as well as consume even infected pork [21]. Considering that tapeworm carriers are at higher risk of consecutive autoinfection resulting in CC [29], this may explain the higher CC seropositivity among men. The CC seropositivity in at least one test was higher among the older age group (>80 years). It has been shown that older populations tend to have a higher proportion of active cysticerci due to a decreasing immune response to infection as one gets older, which facilitates the establishment and maintenance of viable cysticerci [30].

A third of the CC-positive participants, irrespective of whether they also had neurological signs/symptoms such as epileptic seizures and headache, had NCC. Still, the main finding was that epileptic seizures (16%) and headaches (46%) were the most common manifestations. This is consistent with several other studies that have identified epileptic seizures and headaches as the most common manifestation of NCC [3,31,32].

Headaches were ascertained by using a questionnaire. However, we did not collect detailed information regarding the nature, duration, and intensity of the headache, which limits our discussion on headaches. Headaches in NCC are most often recurrent, potentially associated with cerebral inflammatory processes, or chronic, and can be similar to migraines or tension-type headaches but have no specific characteristics [32,33]. The presence of recurrent or chronic headache has been reported as a suggestive finding of NCC in studies conducted in rural areas of Cameroon, Tanzania, and Ecuador [1,34,35]. Most of our participants with NCC had calcified lesions located in the brain parenchyma. The presence of headache in patients with calcified NCC has been demonstrated before in a community setting in Ecuador [36]. Though calcifications are generally considered inactive, they may periodically undergo morphological transformations resulting in transient inflammatory changes in the brain parenchyma. This may also cause recurrent headache episodes mimicking a primary headache disorder [33].

We found that PWEs with NCC were older than PWEs without NCC at the time of investigation. This could be because NCC often manifests clinically only after several years [2,37] and may support the assertion that NCC is one of the most common causes of late-onset epileptic seizures in people with epilepsy [1]. However, there was no significant difference in the age of onset of epileptic seizures or family predisposition to seizures between PWEs with NCC and PWEs without NCC. The former could be due to cultural aspects, as people from rural areas often do not rely on exact ages and may have forgotten about the exact age of seizure onset in the past. The percentage of a positive family history for epilepsy could be expected to be higher in PWEs without NCC, but there are many causes of secondary epilepsy in sub-Saharan Africa other than NCC, such as cerebral malaria and human immunodeficiency virus/acquired immunodeficiency syndrome [38,39], which do not go along with a positive family history. A greater proportion (96%) of lesions in the brains of PWEs with NCC were inactive (calcified). Though the frequency of epileptic seizures is often higher in patients with active/degenerating-stage lesions, they are reportedly common in patients with calcified parenchymal brain cysticerci [11,40]. We also found in our study a good proportion (88%) of participants with calcifications who had never experienced an epileptic seizure. This could be due to the relatively few NCC lesions per patient, the location of the lesion, or merely due to a genetic predisposition to low seizure threshold in people with NCC who develop epilepsy [40].

One in every eight participants with NCC in our study had active lesions only. Under field conditions, participants are mostly asymptomatic, and viable cysts or degenerating parasites are present in only a small minority of people with NCC compared to what is usually seen in hospital settings [11,41]. In the few imaging studies performed in the general population of endemic villages or asymptomatic populations, brain calcifications are more common than viable cysts, suggesting that most cysts degenerate naturally and that major neurological manifestations may not be that frequent [11,41]. This may also explain why there was no difference between participants with NCC and those without NCC regarding neurological findings in our study and highlights the asymptomatic nature of NCC in community settings [11,42].

A limitation of our study is that only serologically positive participants received neuroimaging. The epidemiology of NCC would have been better understood had serologically negative participants been included for CT examination, as previous studies demonstrated the limited accuracy of the serological tests, particularly for people with only calcified lesions [13,43]. This, however, was not possible due to ethical limitations. Another limitation is that during the study period some time elapsed between screening of participants and taking CC-positive participants for CT examination. This is because the CT machine frequently experienced faults and was not operational at all times during the study. Other participants relocated to other villages, while others could not be traced even after follow-up. This, together with the unreached target sample size, could have introduced some selection bias in the study.

## Conclusion

In conclusion, CC and NCC are prevalent in the Chiparamba area of Chipata District in the Eastern Province of Zambia. We demonstrated that patients with NCC are asymptomatic on neurological examination, but the majority present with a history of epilepsy and/or severe headache in community settings. We also demonstrated that calcifications are the most frequent lesions among CC-positive people with NCC among PWEs and people with headache in the villages of Chiparamba community.

The findings of the current study support the need for health education programs targeting *T. solium* transmission, NCC signs and symptoms, prevention, management, and control. Furthermore, targeted public health interventions for surveillance, control, and elimination of *T. solium* infections are required.

## Funding

This study was funded by the German Federal Ministry of Education and Research under CYSTINET-Africa grant numbers 81203604 (CSS) and 01KA1618 (ASW). The funder had no role in the design of the study, data collection, analysis and interpretation, and the writing of the manuscript.

## Ethical approval and statement

Ethical clearance was obtained from the ERES CONVERGE Institutional Review Board (reference number 2018-March-002). Approval was also obtained from the Zambia National Health Research Authority and from the ethics committee of the Klinikum rechts der Isar, Technical University of Munich, Germany, under number 537/18 S-KK. All participants were informed about all aspects of the study before inclusion, and all signed an informed consent form. For individuals below the consenting age (<18 years), written formal consent was obtained from their parents or guardians. The reporting of this study followed the Strengthening the Reporting of Observational Studies in Epidemiology (STROBE) checklist.

## Availability of data and materials

All data generated or analyzed during this study are included in this published article and its supplementary information files.

## CRediT authorship contribution statement

**Gideon Zulu:** Writing – review & editing, Writing – original draft, Visualization, Validation, Software, Resources, Methodology, Investigation, Formal analysis, Data curation, Conceptualization. **Kabemba Evans Mwape:** Writing – review & editing, Validation, Supervision, Resources, Methodology, Data curation, Conceptualization. **Tamara Margarete Welte:** Writing – review & editing, Visualization. **Dominik Stelzle:** Writing – review & editing, Validation, Methodology, Data curation, Conceptualization. **Martin Simuunza:** Writing – review & editing, Software, Resources. **Philipp Prucker:** Writing – review & editing, Formal analysis. **Alex Hachangu:** Writing – review & editing, Investigation. **Wilbroad Mutale:** Writing – review & editing, Supervision. **Maxwell Masuku:** Writing – review & editing, Investigation. **Charlotte Ruether:** Writing – review & editing, Formal analysis. **Chummy Sikalizyo Sikasunge:** Writing – review & editing, Supervision, Resources, Project administration, Methodology, Funding acquisition, Data curation, Conceptualization. **Isaac Khozozo Phiri:** Writing – review & editing, Visualization, Supervision, Resources, Data curation, Conceptualization. **Andrea Sylvia Winkler:** Writing – review & editing, Visualization, Validation, Supervision, Resources, Project administration, Methodology, Funding acquisition, Data curation, Conceptualization.

## Declaration of competing interest

The authors have no competing interests to declare.

## Appendix Supplementary materials

Image, application 1
