# Supplementary material for: Cysticercosis and neurocysticercosis in Eastern Zambia: A cross-sectional, community-based study
Source: IJID Reg. 2026 Sep 21;21:101018. doi: 10.1016/j.ijregi.2026.101018 (PMC13657152; doi:10.1016/j.ijregi.2026.101018)
Supplement: Supplementary file 1 [file mmc1.docx]

**Supplementary material**

Table S1. Cysticercosis seroprevalence by village in Chiparamba of Chipata District.

|  |  | At least one serological test positive | Ag-ELISA positive only | Western blot positive only | Ag-ELISA and Western blot positive |  |
| --- | --- | --- | --- | --- | --- | --- |
|  |  | N (%) | N (%) | N (%) | N (%) | p-value ^¥^ |
|  | 2876 | 254 (8·8) | 70 (2·4) | 149 (5·2) | 35 (1·2) |  |
| Bvuso | 96 | 7 (7·3) | 3 (3·1) | 2 (2·1) | 2 (2·1) | 0·001 |
| Chiweteka | 221 | 18 (8·1) | 2 (0·9) | 14 (6·3) | 2 (0·9) |  |
| Chimutangati | 31 | 0 (0·0) | 0 (0·0) | 0 (0·0) | 0 (0·0) |  |
| Chinthona | 86 | 11 (12·8) | 3 (3·5) | 7 (8·1) | 1 (1·2) |  |
| Chuni chikuwe | 91 | 3 (3·3) | 1 (1·1) | 1 (1·1) | 1 (1·1) |  |
| Chiweza | 132 | 5 (3·8) | 3 (2·3) | 1 (0·8) | 1 (0·8) |  |
| Kaliyoyo | 77 | 3 (3·9) | 1 (1·3) | 1 (1·3) | 1 (1·3) |  |
| Kanamanja | 146 | 15 (10·3) | 1 (0·7) | 11 (7·5) | 3 (2·1) |  |
| Kasosa | 120 | 14 (11·7) | 2 (1·7) | 9 (7·5) | 3 (2·5) |  |
| Kabendama | 168 | 20 (11·9) | 2 (1·2) | 15 (8·9) | 3 (1·8) |  |
| Kochiwe | 58 | 10 (17·2) | 6 (10·3) | 2 (3·4) | 2 (3·4) |  |
| Kalumekalinga | 153 | 12 (7·8) | 2 (1·3) | 7 (4·6) | 3 (2·0) |  |
| Lufu | 145 | 8 (5·5) | 0 (0·0) | 5 (3·4) | 3 (2·1) |  |
| Majuku | 75 | 3 (4·0) | 0 (0·0) | 3 (4·0) | 0 (0·0) |  |
| Misholo | 168 | 12 (7·1) | 4 (2·4) | 8 (4·8) | 0 (0·0) |  |
| Mkanile | 99 | 8 (8·1) | 4 (4·0) | 3 (3·0) | 1 (1·0) |  |
| Mteyo | 83 | 3 (3·6) | 2 (2·4) | 0 (0·0) | 1 (1·2) |  |
| Mulilo | 175 | 24 (13·7) | 8 (4·6) | 14 (8·0) | 2 (1·1) |  |
| Mzamo | 57 | 9 (15·8) | 3 (5·3) | 6 (10·5) | 0 (0·0) |  |
| Nkhunda | 86 | 4 (4·7) | 2 (2·3) | 2 (2·3) | 0 (0·0) |  |
| Payani | 28 | 3 (10·7) | 0 (0·0) | 3 (10·7) | 0 (0·0) |  |
| Simeon | 147 | 11 (7·5) | 4 (2·7) | 6 (4·1) | 1 (0·7) |  |
| Soweto | 128 | 11 (8·6) | 3 (2·3) | 6 (4·7) | 2 (1·6) |  |
| Vwala | 184 | 29 (15·8) | 8 (4·3) | 19 (10·3) | 2 (1·1) |  |
| Yohane | 122 | 11 (9·0) | 6 (4·9) | 4 (3·3) | 1 (0·8) |  |

Ag, antigen; ELISA, enzyme-linked immunosorbent assay. ^¥^ p-value for the comparison of village with the variable “at least one serological test positive.”

Table S2. Predictors of at least one positive cysticercosis serological test result (Ag-ELISA or Western blot).

| **Predictor variable** |  | **OR** | **95% CI** | **p-value** |
| --- | --- | --- | --- | --- |
| Sex | Male | 1·5 | 1·1–1·9 | 0·02 |
|  | Female | Ref |  |  |
| Age | 10–20 | Ref |  |  |
|  | 21–40 | 1·1 | 0·8–1·5 | 0·53 |
|  | 41–60 | 1·3 | 0·9–1·9 | 0·12 |
|  | 61–80 | 1·2 | 0·7–2·1 | 0·57 |
|  | 81–100 | 4·1 | 1·6–10·6 | 0·004 |

OR, Odds ratio; Ref, Reference

Table S3. Neurological examination, summary of abnormal findings.

| **Neurological examination** | **Abnormality** | **Overall**  **n (%)** | **NCC**  **n (%)** | **No NCC**  **n (%)** |
| --- | --- | --- | --- | --- |
|  |  | **236** | **80** | **156** |
| Cranial nerve abnormalities | Visual acuity only | 15 (6·4) | 6 (7·5) | 9 (5·8) |
|  | Visual fields only | 1 (0·4) | 0 (0·0) | 1 (0·6) |
|  | Visual acuity and visual fields | 4 (1·7) | 2 (2·5) | 2 (1·3) |
|  | Hearing impairment | 6 (2·5) | 2 (2·5) | 4 (2·6) |
|  | Dysphagia | 1 (0·4) | 0 (0·0) | 1 (0·6) |
| Muscle strength deficits | Hemiparesis | 2 (0·8) | 1 (1·3) | 1 (0·6) |
| Muscle tone abnormalities (hypertonia) |  | 2 (0·8) | 1 (1·3) | 1 (0·6) |
| Primitive reflexes | Palmomental reflex | 1 (0·4) | 0 (0·0) | 1 (0·6) |
| Deep tendon reflex abnormalities | Hyperactive | 3 (1·3) | 2 (2·5) | 1 (0·6) |
| Extrapyramidal abnormalities | Dyskinesia, akinesia, dystonia | 0 (0·0) | 0 (0·0) | 0 (0·0) |
| Gait abnormalities | High-stepping | 1 (0·4) | 1 (1·3) | 0 (0·0) |
|  | Hemiplegic | 1 (0·4) | 0 (0·0) | 1 (0·6) |
|  | Antalgic (limp) | 1 (0·4) | 1 (1·3) | 0 (0·0) |
|  | Unable to walk or stand | 1 (0·4) | 1 (1·3) | 0 (0·0) |
| Coordination abnormalities | Finger-nose test | 2 (0·8) | 1 (1·3) | 1 (0·6) |
|  | Knee-heel test | 1 (0·4) | 1 (1·3) | 0 (0·0) |
| Sensation abnormalities | nil | nil | 0 (0·0) | 0 (0·0) |
| Mental state abnormalities | Appearance | 1 (0·4) | 1 (1·3) | 0 (0·0) |
|  | Behaviour | 2 (0·8) | 1 (1·3) | 1 (0·6) |
|  | Cognition | 2 (0·8) | 1 (1·3) | 1 (0·6) |

NCC, Neurocysticercosis; n, Number

Table S4: Abnormal radiological findings in participants without neurocysticercosis.

| **Age**  **(Years)** | **Sex** | **CT findings** | **Clinical findings** |
| --- | --- | --- | --- |
| 46 | M | Global atrophy | Nil |
| 12 | F | Two non-specific calcifications parafalcine and frontobasal | Nil |
| 23 | F | Cerebellar atrophy | Nil |
| 14 | M | Most likely postischemic defect with laminar necrosis left parietal and occipital | Epileptic seizures |
| 54 | F | Non-specific left frontal calcification | Nil |
| 45 | M | Cerebellar atrophy | Hypertensive |
| 19 | F | One non-specific parafalcine and parietal calcification left | Epileptic seizures |
| 60 | M | Postischaemic scar in left basal ganglia | Nil |
| 40 | M | Small non-specific calcification left parietal lobe | Nil |
| 22 | M | Most likely postischemic defect in left frontal lobe | Epileptic seizures |
| 19 | M | Atrophic left hemisphere, partly with malacic liquor filled defects and ex-vacuo dilatation of the left lateral ventricle, hyperplastic and left accentuated sinus frontalis and thickening of the left skull | Epileptic seizures, cognitive impairment, right hemiparesis (Possibly Dyke-Davidoff-Masson syndrome) |
| 20 | M | Most likely postischemic defect left parietal lobe | Bilateral deafness |

CT, Computed tomography; M, Male; F, Female
